# Family assessment conversations as a tool to support families affected by parental mental illness: a retrospective review of electronic patient journals

**DOI:** 10.1186/s13033-018-0199-x

**Published:** 2018-04-24

**Authors:** Camilla Lauritzen, Anne Berit Kolmannskog, Anette Christine Iversen

**Affiliations:** 1 Regional Center for Child and Youth Mental Health & Child Welfare (RKBU North), UiT- Arctic University of Norway, 9037 Tromsø, Norway; 2 Department of Health Promotion and Development, Faculty of Psychology, University of Bergen, Bergen, Norway

**Keywords:** Parental mental illness, Family assessment

## Abstract

**Background:**

Previous research has shown a link between parental mental illness and adverse development in their offspring. In Norway, it is mandatory for health professionals to identify if patients in adult mental health services have children, and subsequently to provide support for the children. An important tool to detect if families are affected by parental mental illness and to assess if there is a need for further intervention is the Family Assessment Conversation. Family Assessment Conversations is potentially a powerful tool for communication with families affected by parental mental illness because it facilitates early identification of children at risk of various adversities due to the family situation. Additionally the tool may initiate processes that enable children and parents to cope with the situation when a parent becomes seriously ill. Little is however known about how the mental health practitioners use the family assessment form in conversations, and to what extent they record relevant information in the electronic patient journals.

**Methods:**

The main aim of the study was to provide information about the existing practice within mental health services for adults in terms of parental mental illness and family assessment conversations. The project is a retrospective journal review. The data base consists of relevant journal data from 734 patients aged 20–60 years admitted. In total, 159 recordings of family assessment conversations were discovered.

**Results:**

The main result in this study was that many of the questions in the family assessment form lacked documented responses and assessments from the healthcare professionals. Only 17% of the participants had been assessed with the total family assessment form. Additionally, there was a lack of documentation about whether or not the children had been informed in a large proportion of the assessment forms (31%). A total of 55% say that the child has not been informed. This implies that there is still a long way to go in order to make sure that children of parents with a mental illness are given relevant information and support.

**Conclusions:**

The documentation and family assessment frequency is low and reflects the challenges healthcare professionals and patient experience when the child’s situation becomes the topic of assessment. There is a need to further investigate the challenges of changing the mental health systems to incorporate the children and families of patients. More research should promote knowledge on what may facilitate family assessment dialogue.

## Background

Previous research has shown a clear connection between parental mental illness and the child’s daily functioning [1–3]. Consequences for the child may include developmental disorders, mental illness, economic and social problems, as well as childhood neglect, which in some cases leads to relocation from the home [4, 5]. In addition, the child itself may be put in the position of taking a caregiving role in the family [6]. Children want the best for their parents and, in serious and long-term illness, often take on major caregiving tasks in order to make the family life to run normally [6]. These tasks may in many cases exceed the child’s own well-being, schooling and leisure time [4, 5].

In a literature review from 2015, Grové et al. concluded that it is not the parental mental illness in itself that is problematic for the child. There are several risk factors related to parental mental illness that influence the outcome for the child. Reduced parenting capacity, lack of social support, stigma and discrimination, housing shortage and poverty, as well as coincidence of substance abuse or violence are all factors that, together with mental illness, create difficulties for the child [7].

Parents with mental illness and their children constitute the most vulnerable families in society [8]. In such families, the adults generally have poor health, economic problems and lower social status than in non-mental illness families. These factors can collectively and/or individually increase the risk of affecting the children negatively genetically, psychologically and environmentally [8, 9]. Knowledge about how mental illness affects parenting may moderate the adverse outcomes of parental mental illness. Having a realistic perception of the family’s situation and the parents’ illness is considered a protective factor for the children [10]. Based on this knowledge, there is an under-developed potential for preventing serious difficulties for the children of parents affected by parental mental illness through providing information about mental illness.

In many countries, this prevention potential has been taken into account recently, and alterations to health legislation and health procedures have been made. In countries like Finland and Sweden it is mandatory to assess if patients have children and to act upon this knowledge. In Norway, healthcare professionals are required to assess if patients have children, and if so, whether the child should be given information or support. They are also to provide information and guidance to the patient (or an alternative person who cares for the child) about the consequences of parental mental illness [11]. The purpose of the Norwegian health legislation is, according to the Directorate of Health, “to ensure that children are identified early and that processes are initiated that enable children and parents to cope with the situation when a parent becomes seriously ill.” Health personnel are also required by law to report to child welfare and protection services if they have reason to believe that a child is maltreated or neglected at home (The Health Personnel Act § 33). Norwegian child welfare system is child-focused and family oriented and most children receive in-home services such as parenting counselling, support person, week-end home etc. [12]. These services can moderate the effect of additional risk factors such as lack of social support and reduced parenting capacity. It is only when assistance at home is not sufficient to generate satisfactory conditions that children may be taken in out of home care either as a voluntary placement in agreement with the parents or by a care order.

Providing information to children and families about parental mental illness may be challenging. Many studies have shown that health professionals would like to have more knowledge and skills about conducting such conversations with children and families [4, 13–15]. In a previous study of assessment routines in Norway, it was documented that patients to a large degree were asked if they have children, but the responses were not satisfactorily recorded in the electronic patient journal (EPJ) despite this being part of the health authorities’ procedures [4]. The lack of knowledge and skills among healthcare professionals may be an important explanation to the lack of registration. According to Ruud et al., there is a correlation between the degree of training in EPJ registration and actual registration [4].

Family Assessment Conversations is potentially a powerful tool for communication with families affected by parental mental illness because it facilitates early identification of children at risk of various adversities due to the family situation. Additionally the tool may initiate processes that enable children and parents to cope with the situation when a parent becomes seriously ill. Person and Benzein argues that family conversations may support family health [13]. According to their study, family conversations facilitates a process in which family members develop an increased understanding of themselves and others and of their interactional patterns [13].

According to Norwegian legislation, a family assessment conversation is to be offered to all patients who have minor children. The conversation is to be documented in the patient’s journal. As a support to the health care personnel that is to carry out these conversations, Norwegian authorities developed a family assessment form which was published just after the new legislation became effective in 2010 [11]. Norwegian hospitals have since adopted the family assessment form into their procedures. The form consists of a questionnaire that health professionals use in the family assessment conversation with the patient. The family assessment form contains 30 questions. The questions may be answered with Yes or No, or as free text. Several of the questions investigate the family composition and the patient’s relationship with the child. Secondly, there are questions about cooperation with other agencies, and the patient’s own strategies to protect the child. A more detailed description of the family assessment form is included under *measures* in the “Methods” section.

The healthcare provider has the opportunity to inform the parents and the child about consequences of parental mental illness using the family assessment conversation as a tool. The healthcare personnel should therefore ask whether the child has been informed and if the patient agrees that information about the patient’s disease may be given to the child by the healthcare personnel. A second family conversation to follow up the child is then planned with the parents alone in advance. In order to carry out such conversations, consent is required from the patient to contact other adults who may be responsible for the child while the patient is admitted. Little is known about how mental health practitioners use the family assessment form in conversations, and to what extent they record relevant information in the EPJ.

## Methods

### Aims of the study

The main aim of the study was to provide information about the existing practice within mental health services for adults in terms of parental mental illness and family assessment conversations. The study was designed to evaluate the following research questions:

1. Which information do health personnel record in the EPJ based on the family assessment conversations?
2. Do health personnel inform the child’s school, public health nurse or kindergarten about the family situation?
3. Are other agencies involved to assist the family?
4. Have the parents given consent to contact other agencies?
5. Has the child received information about the parent’s mental illness?
6. Have the parents received materials to assist informing the child in this situation?
7. Did the health personnel consider the child’s need for information?
8. Did the health personnel conduct conversations with the family?
9. Did the health personnel report concerns to the child welfare and protection services about the child?

### Participants

This study is a retrospective review of electronic patient journals (EPJ) of patients within adult mental health services. The data was collected at two different public psychiatric hospitals in Norway. A total of 811 electronic patient journals were reviewed.

### Procedure

As the purpose of this project was to investigate whether a family assessment conversation had been conducted, patients in the age group 18–60 years were elected. All journals from 2010, 2012 and 2014 were reviewed. The year 2010 was chosen as baseline since Family assessment forms were initially implemented 2010. Given the resources available for the project, we did not have the capacity to assess all admissions between 2010 and 2015. We therefore selected the years 2012 and 2014 to see if the implementation had led to a changed practice. 2012 and 2014 provide adequate interval to answer our research questions. From August 2015 a revised Family assessment forms was implemented. All Family assessment forms registered in the 811 journals were extracted, a total of 159. Demographic data, such as diagnosis and cause of hospital admission, was not retrieved at the patient level for confidentiality reasons. For each patient journal, the document type “Family assessment form” and “Family follow-up” was selected. All collected data were registered as indirectly identifiable information (Health Registry Section 2b). Information that could directly identify the patient was omitted in the transfer of data from the EPJ to SPSS. Initially, 170 family assessment forms were found. There were 11 forms that had been registered twice since the patient was admitted both in 2012 and 2014. These were reviewed and deleted. A total of 159 family assessment forms are included in the selection. The number of unique survey forms (159) does not correspond to the number of patients surveyed, which is 123. At the time of the assessment, the patients’ children were in the age group 0-18 years.

### Measures

#### Family assessment form

The family assessment form consists of five groups of questions. The form is filled out by the health care personnel during or after the family assessment conversation. The information the patient provides during the conversation is then recorded in the EPJ as a family assessment form. The five groups of questions are as follows:

#### Children of patients

How many children/step-children are there under 18 years old in the family? How many children live with the patient? Recordings of the children’s name, age, school/daycare facility, siblings and names of other caregivers.

#### Network

Where does the child live while the parent is in hospital? Who takes care of the child? Does the family have someone who helps out? Is the kindergarten, school, public health service or school nurse informed about the situation? Are the child welfare service involved? Are other agencies involved, such as:, mental health services for children, the family’s general practitioner etc.?

#### Needs

What are the patient’s thoughts about the consequences for the children? Does the child have questions, if so what would they like to know? Has the parent or other adults observed changes in the child? Is the parent concerned about the child’s situation? Would the parent like help from others? Are there people in the family’s network that can provide support for the family?

#### Information

What does the child know about the situation of the parent? How does the parent want the information to the child to be done? What materials to support information has been handed out to the parent? Does the parent give consent to provide information and further follow-up to the child? Have appointments been made to see the child?

#### The assessment conclusions of the health personnel

Is the children’s need for information and further follow-up satisfactory taken care of? Is there a need for further action in terms of follow-up? Might the children need health care? Has a notification of concern been given to the child welfare and protection services, if relevant?

### Analyses

Selected parameters were encoded and transferred to SPSS (IBM SPSS Statistics Version 24). After all data was uploaded to SPSS, data was reviewed and corrected for errors and deficiencies using frequency analysis. All descriptive analyses were conducted in SPSS on the anonymous data. The SPSS file contained 34 categorical variables where all (except one) were numeric and nominal. The SPSS file is available for details about the variables.

The frequency analysis based on data from the SPSS file does not provide the number of patients. Many patients are registered with more than one admission. A formula was therefore prepared in EXCEL to re-calculate the number of admissions to the number of patients based on the code key for each patient. Statistical analyzes consisted of frequency analysis (number), percentage distribution and Chi square tests. The calculator used for Chi square calculations was obtained from the internet, http://www.socscistatistics.com/Default.aspx.

## Results

A total of 811 patient records were reviewed. After the data set was prepared for analysis, the total number of patients included was 734. The majority of the patients were men, and the majority of the sample was between 30 and 50 years old. A total of 57% of the sample had been asked if they had children 0–18 years old. Out of these 734, only 122 (17%) had been assessed with the total family assessment form. The information that was recorded in the EPJ about the families that were assessed is presented in Table 1. As can be seen from Table 1, only 43% of the patients lived with the child.

**Table 1:** Which information do health personnel record in the EPJ based on the family assessment conversations?

	Number of family assessment forms (n = 159)	Number of patients (n = 122)
Gender		
Female	78 (49%)	56 (45%)
Male	81 (51%)	66 (55%)
Age		
20–30	14 (9%)	14 (12%)
30–40	67 (42%)	49 (40%)
40–50	65 (41%)	49 (40%)
50–60	13 (8%)	10 (8%)
Year admitted		
Admitted 2010	Missing data	Missing data
Admitted 2012	46 (29%)	39 (32%)
Admitted 2014	113 (71%)	83 (68%)
EPJ		
Registered with children	98 (62%)	69 (57%)
Registered without children	7 (4%)	7 (6%)
Not registered	54 (34%)	46 (38%)
Family assessments		
Assessed 2010	Missing data	Missing data
Assessed 2011	11 (7%)	9 (7%)
Assessed 2012	37 (23%)	37 (25%)
Assessed 2013	21 (13%)	20 (13%)
Assessed 2014	65 (41%)	58 (38%)
Assessed 2015	25 (16%)	25 (17%)
Contact		
Lives with child	68 (43%)	56 (43%)
Does not live with child	91 (57%)	74 (56%)
Children		
Number of children	197	161
Age of children	Missing data	Missing data

An important part of the work of identifying and following up children of patients is about having a good cooperation with arenas the child attends. An overview of the extent to which the schools, kindergartens or public health nurses had been informed about the child’s situation is presented in Table 2. The school was the most frequently mentioned arena among the patients in the sample, 18% of the assessment forms contained evidence that the school had been oriented. The other arenas each had a low documentation frequency and are combined in the table under Others. However, 40% stated that neither of the aforementioned instances had been informed or involved with the child or the family.

**Table 2:** Is the kindergarten/school/public health nurse/school nurse informed about the situation?

	Family assessment form (n = 159)	Patients (n = 142)
School	28 (18%)	23 (16%)
Other^a^	10 (6%)	10 (11%)
All	8 (5%)	8 (21%)
None	46 (29%)	43 (30%)
Not registered	67 (42%)	58 (52%)

^a^Kindergarten, public health nurse, school nurse

We also wanted to see if there were any recordings of other arenas who were involved to support the family. The external services that the child and the family have or may be in contact with included child welfare and protection services, general practitioners and child mental health services. Child welfare and protection services were involved in 38 of the patients’ family lives. The other services each had a lower documentation frequency and are combined in the table under Others (Table 3).

**Table 3:** Are other agencies involved to assist the family (e.g., CWPS, GP, CAMHS)

	Family assessment form (n = 159)	Patients (n = 142)
CWPS	54 (34%)	45 (32%)
Other^a^	21 (12%)	20 (14%)
All	9 (6%)	8 (6%)
None	38 (24%)	13 (24%)
Not registered	37 (23%)	26 (24%)

^a^GP, CAMHS

In terms of parental consent to contact other agencies, there was only documented consent in 8% of the patients that were assessed. Table 4 shows that a total of 50% of the patients did not consent to the health care workers contacting other agencies. Documentation is missing in 40% of the assessments regarding whether or not consent had been obtained (Table 4).

**Table 4:** Have the parents given consent to contact other agencies?

	Family assessment form (n = 159)	Patients (n = 136)
Yes	13 (8%)	11 (8%)
No	79 (50%)	70 (52%)
Not registered	67 (42%)	55 (40%)

According to Norwegian legislation, children of mentally ill patients have the right to receive information about the situation when a parent is ill. Table 5 presents information about whether the children have received information about the parent’s mental illness. There is a lack of documentation about whether or not the children have been informed in a large proportion of the assessment forms (31%). A total of 55% say that the child has not been informed (Table 5).

**Table 5:** Has the child received information about the parent’s mental illness?

	Family assessment form (n = 159)	Patients (n = 140)
Yes	48 (31%)	41 (30%)
No	61 (38%)	55 (39%)
Not registered	45 (31%)	44 (31%)

We compared information about whether or not the child has been informed of the disease and the parents’ consent to follow-up and we found a significant connection. A Chi square test was conducted: (n = 159) = 37.42, p = 0.00. Among patients who consented to informing the child, and believed that the child did not know about the parents’ disease, 44% lived together with the child. Among those who did not agree to providing information, and at the same time stated that the child did not know about the disease, 61% lived together with the child.

One of the main purposes of the assessment is the opportunity it provides to inform the patient about what the child needs when a parent is affected with parental mental illness. Table 6 gives an overview of the number of people who have received materials consisting of information about parental mental illness in writing.

**Table 6:** Have the parents received materials to assist informing the child in this situation?

	Family assessment form (n = 159)	Patients (n = 136)
Yes	33 (21%)	29 (21%)
No	35 (22%)	33 (24%)
Not registered	91 (57%)	74 (55%)

More than half of the assessment forms lacked documentation of whether or not material had been distributed. A total of 21% of the patients had been given material, while as 24% had not had any material distributed. A Chi square test showed a significant association between location and distributed information material, (n = 765) = 12.07, p = 0.00. One of the participating hospitals had a higher documentation frequency for distributed material than the other. Hospital 1 had distributed materials in 33% of the cases, while as for the second hospital this was only done in 13% of the cases. Both hospitals had a high proportion where they had not documented whether or not such materials had been distributed, 54% in hospital 1 and 59% in hospital 2.

When asked if the children were informed and safeguarded to a satisfactory degree, the health personnel failed to provide their personal view in 60% of the cases. In 28% of the cases, they perceived the situation of the child as being taken care of, and in 12% of the cases they stated that the child’s situation was not sufficiently taken care of. See Table 7 for details about health personnel’s consideration of the children's need for information.

**Table 7:** Did the health personnel consider the child’s need for information?

	Family assessment form (n = 159)	Patients (n = 139)
The child’s need for information was considered		
Yes	45 (28%)	41 (29%)
No	19 (12%)	18 (13%)
Not registered	95 (60%)	80 (58%)
In need of follow-up?		
Yes	37 (23%)	32 (23%)
No	38 (24%)	36 (26%)
Not registered	84 (53%)	71 (51%)

Another important aspect in the Norwegian legislation about parental mental illness is family conversations to provide relevant information about the consequences of parental mental illness and to assess needs for further assistance. Table 8 provides an overview of planned and conducted family conversations.

**Table 8:** Did the health personnel conduct conversations with the family?

	Family assessment form (n = 159)	Patients (n = 141/128)
Planned conversation		
Yes	21 (13%)	21 (15%)
No	61 (39%)	55 (39%)
Not registered	77 (49%)	65 (46%)
Conducted conversation		
Yes	13 (8%)	13 (10%)
No	0	0
Not registered	146 (92%)	115 (90%)

In terms of conversations with the families, there were 15% of the patients giving consent. Only 10% had registered conducted family conversations in their journals. A total of 39% of the patients did not consent to such conversations, and for 46% of the patients there are no records. When it was documented that the patient had accepted a family conversation, we also looked into to what extent the family conversation had been conducted. We found that 69% of the agreed conversations were also documented in the patient’s journal. A Chi square test was conducted: (n = 159) = 38.18, p = 0.00. There were no records of conversations having been performed in spite of a lack of consent.

Another important aspect of Norwegian legislation is about the obligation to report concerns to the child welfare and protection services if there are any. The final entry in the assessment form contains questions about the need for a notification to the child welfare and protection services. Among the 159 assessment forms included in our sample, only six cases were registered where a notification to the child welfare and protection services was seen as necessary. This equals 4% of the total assessment forms. In 16% of the forms, health professionals documented that there was no need for concern. In 80 percent, health professionals have not documented their assessment of the need for reporting concerns to the child welfare and protection services.

## Discussion

The purpose of the family assessment is to provide health professionals with an overview of the child’s situation, to assist the parents with information, and to support parenting, cf. the Health Personnel Act Section 10a. The family assessment conversation thus affects several aspects of the patient and the child’s life.

A consistent finding in this study was that many of the questions in the family assessment form lacked documented responses and assessments from the healthcare professionals. How often the healthcare provider left the answer options open varied from question to question and from patient to patient. The degree of documentation can therefore be said to reflect the challenges the healthcare staff and the patient experience when the child’s situation becomes a discussion theme. However, we do not know to what extent the health personnel finds the assessment form feasible. The form has not been tested systematically in the field of practice. When implementing new routines it is important to conduct a process evaluation to understand the challenges practitioners may face [16]. Given the low documentation grade in the present study, we believe there is need to understand health personnel’s perception and experience of the assessment form. Research from the child protection field shows that it is not straightforward to talk about parenting in an environment where the purpose of the interview is both controlling and informative [17]. Good communication is based on trust and knowledge of the role of the parties in a conversation. The patient and health care personnel are not equal parties [18]. It will therefore be crucial for a trustworthy cooperation that both parties are able to convey their message within the framework the hospitalization provides them. The patient’s concerns about themselves and their children, and the health personnel’s assessments of the patient and the child’s situation, will have an impact on the cooperation.

According to Statistics Norway’s database, there is an overweight of parents with mental illness and substance abuse problems among children receiving support from the child welfare and protection services [19]. In our sample, the child welfare and protection services was the most frequently mentioned agency (40%) among patients when they were asked about which additional services they are in contact with. Of the families receiving support from the child welfare and protection services, 33 per cent also stated that they have custody of their children. This may indicate that the family receives some kind of in-home service. This is not in accordance with the survey for Ruud et al. from 2015 where a lower percentage of patients having custody was recorded. However, they did find that patients in adult mental health services received more help from child and adolescent psychiatry as well as child welfare and protection services than other patient groups [4]. Within adult mental health services, 10% of the patients and respectively 15% of the health care personnel stated that families received assistance from the child welfare and protection services [4]. However, Ruud et al. pointed out that patients actually get less help than health professionals believe and that this may result in too few notifications of concern to the CWPS. Ruud also showed that healthcare professionals may have limited insight into what the family actually receives [4]. This may be the case in our study as well.

In our sample, many patients stated that no other services or agencies were involved (24%), and 70 percent of these patients also stated that they still live together with their children. In total, 20 percent of the patients stated that the school was informed of their illness. Previous research has shown that there is a different view of the need for external support among healthcare professionals, parents and children. The children themselves emphasize friends as their main source of external support, while the parents emphasize school as an important arena for support for the children. Based on this, 20% seems to be a very low number [4, 13, 18–21].

The healthcare personnel are obligated to obtain consent to inform schools, public health nurses or other services about the children’s situation, but only a few (8%) in our sample gave the healthcare personnel such consent. The healthcare personnel have not documented the patient’s response to the consent question in 42% of the conversations. As mentioned initially, healthcare professionals cannot provide health information about the patient to others without a legal basis in the form of consent from the patient [11]. The health personnel are bound by confidentiality. However, confidentiality does not have to hinder cooperation around a child’s situation. The Directorate of Health emphasizes in its guidance that it is possible to collaborate on the child’s situation without passing on information about the parent’s health [11].

In the family assessment conversation, one of the questions to the patient is whether the child has been informed about the parent’s illness. It is primarily the parents who are responsible for providing the child with adequate information, and it is therefore natural for the healthcare staff to support the patient in providing this information in a good way to the child [22]. In this study we found that approximately 30 percent of the children are informed of the parents’ illness. However, we are missing documentation on whether the child is informed in an additional 30 percent of the cases. This means that in a third of family assessment conversations there is no information about the child’s knowledge of the parent’s illness. There may be many reasons why so many people do not provide information about this. The child may be too young for the question to be relevant, or the parents may not live together with the child themselves. About 50 percent of the patients that have been asked this question did however state that the child had not been informed. This corresponds to approximately 85 children in this sample, showing that a significant proportion of children lack adequate knowledge of their parent’s illness. Of the parents who stated that the child had not been informed, half of them also stated they had not received information from health professionals about the importance of information for the child’s well-being and daily functioning. These results are in correspondence with previous research showing that there is a discrepancy between what the healthcare staff state to have informed about and what the patient and the children state they have received information about. In the study of [4], 40 percent of the patients reported that they had received information, while 60 percent of the health personnel stated that they had provided this information [4].

A total of 15 percent of the patients who completed the initial family assessment conversation agreed to the follow-up conversation with the child. However, only 69 percent of the agreed talks are also documented in the patient journal which may indicate that they were not completed as intended. There may be many reasons for this. One reason may be that it takes time to arrange such a meeting and that the patient has meanwhile been moved to another department or discharged. Another explanation may be that the children themselves do not want such a conversation [7]. In our sample, 39 percent of the patients declined the follow-up conversation with the children. The intention of the Health Personnel Act is that all children should be identified and registered in the patient’s journal, but the follow-up conversations between the health care personnel and the families are not obligatory. Only when patients and therapists consider it appropriate should the child be invited to a conversation with healthcare professionals [4].

In the final section of the family assessment form, the healthcare personnel is asked to make an independent evaluation of the child’s situation based on the information provided in the assessment conversation. They are to consider if the child’s need for information and follow-up has been met. We found that the documentation frequency of these questions was low. This assessment of the child’s situation was only accounted for in 54 of the 159 assessment forms. In 75 of 159 forms it was documented whether or not there was a need for further follow-up.

Ruud et al. [4] suggested that the legislation can give healthcare professionals and parents a superficial understanding of what providing information for children means. If the health personnel do not have sufficient knowledge of the consequences of parental illness for the child, they may over-estimate the ability of the patient and the child to handle the situation [4].

There may be other explanations to why so many health professionals did not document their assessments. Sometimes the conversations may have been initiated, but not been completed for various reasons. Perhaps there has been a need to discuss with a colleague and documenting the final assessment of the situation may have been forgotten, or it may be due to uncertainty. Lines et al. [23] showed that healthcare professionals in many cases lacked sufficient knowledge about how children’s conditions at home can be detected and followed up. This lack of knowledge may cause hesitation in terms of notifying the child welfare and protection services when they are concerned about the child [23]. In our study, only 6 of 159 entries about reporting concern was detected. However, in 63 of 159 entries child welfare and protection services was already involved. Lines [23] emphasize that healthcare professionals, and especially the nurses, are well informed of the legal and ethical responsibility they have to notify the child welfare and protection services in serious cases. The authors believe this insecurity is grounded in a sense of lack of support and information from coworkers and executives. Uncertainty, in many cases, leads to a lack of reporting [23]. Lines argues that nurses need to have sufficient knowledge and skills to take responsibility when children are at risk [23].

It is first and foremost the parents who are to be the primary source of information to the child. The role of the healthcare personnel in supervising the parents may thus be influenced by how the patient perceives the staff; as an inspector or an auxiliary. This dilemma is well known within child welfare work and research literature provides indications that it is a complex interaction that requires openness, warmth and understanding, as well as honest and correct information [24, 25].

Venables et al. referred to several international studies showing that parents feel fear, helplessness and stigmatization in the face of child welfare and protection services [25]. Studsrød et al. reported that most parents understood the reason for referral to child welfare service as wanting to help the child or as a mandatory duty [26]. Recognizing that there are competing considerations between the needs of the children and the parents is important for anyone working with vulnerable families [27]. Taking the child’s standpoint is a moral, political and ideological standpoint in our Western culture, set forth in, among others, the Children’s Convention [15]. Choosing between the adult perspective and the child perspective often puts us in difficult dilemmas [28].

### Study limitations

The study design is a retrospective review, and such studies depend on the quality of the data already recorded in the journal and how the data extraction is carried out from the journals to the quality register. The research questions are therefore dependent on the recordings and cannot be freely chosen.

## Conclusions

The review of the electronic patient journals suggests that the routines to safeguard children of mentally ill parents have been challenging to implement in daily practice.

The documentation and family assessment frequency is low. Only 17% of the participants had been assessed with the total family assessment form. Additionally, there was a lack of documentation about whether or not the children had been informed in a large proportion of the assessment forms (31%). A total of 55% say that the child has not been informed. This implies that there is still a long way to go in order to make sure that children of parents with a mental illness are given relevant information and support.

There is a need to further investigate the challenges of changing the mental health systems to incorporate the children and families of patients.

## Abbreviations

EPJ: Electronic patient journal
CWPS: Child welfare and protection services
CAMHS: Child and adolescent mental health services
GP: General practitioner
NSD: Norwegian data protection officer
